# In vivo effectiveness and safety of probiotics on prophylaxis and treatment of oral candidiasis: a systematic review and meta-analysis

**DOI:** 10.1186/s12903-019-0841-2

**Published:** 2019-07-10

**Authors:** Lijun Hu, Mimi Zhou, Andrew Young, Weiwei Zhao, Zhimin Yan

**Affiliations:** 1 Department of Oral Medicine, Peking University School and Hospital of Stomatology, 22 South Zhongguancun Avenue, Haidian District, Beijing, 100081 People’s Republic of China; 2 Department of Diagnostic Sciences, Arthur A. Dugoni School of Dentistry, University of the Pacific, 155 Fifth Street, San Francisco, CA USA

**Keywords:** Oral candidiasis, Probiotics, Effectiveness, Safety, Clinical trial, Mouse animal model

## Abstract

**Background:**

To systematically review and assess the in vivo effectiveness and safety of probiotics for prophylaxis and treating oral candidiasis.

**Methods:**

A literature search for studies published in English until August 1, 2018 was conducted in the following databases: PubMed, EMBASE, Cochrane Library, and Web of Science. Randomized controlled clinical trials and experimental mouse animal model studies comparing probiotics (at any dosage and in any form) with control groups (placebo, blank control or other agents) and reporting outcomes of the prophylactic and therapeutic effects were considered for inclusion. A descriptive study and, potentially, a meta-analysis were planned.

**Results:**

Six randomized controlled clinical trials and 5 controlled experiments of mouse animal models were included in the systematic review. Four randomized controlled clinical trials comparing a probiotics group with a placebo/blank control group in 480 elderly and denture wearers were included in the meta-analysis. The overall combined odds ratio of the (random effects) meta-analysis was 0.24 (95% CI =0.09–0.63, *P* < 0.01). The overall combined odds ratio of the (fixed effects) sensitivity analysis was 0.39 (95% CI =0.25–0.60, *P* < 0.01) by excluding a study with the smallest sample size. These analyses showed that there was a statistically significant difference in the effect of probiotics compared with the control groups in elderly and denture wearers. The remaining 2 studies compared probiotics with other agents in a population aged 18–75 years and children aged 6–14 years respectively, and were analyzed descriptively. Meta-analysis and descriptive analyses indicated that probiotics were potentially effective in reducing morbidity, improving clinical symptoms and reducing oral *Candida* counts in oral candidiasis. The biases of the included studies were low or uncertain. The relatively common complaints reported were gastrointestinal discomfort and unpleasant taste, and no severe adverse events were reported.

**Conclusions:**

Probiotics were superior to the placebo and blank control in preventing and treating oral candidiasis in the elderly and denture wearers. Although probiotics showed a favorable effect in treating oral candidiasis, more evidence is required to warrant their effectiveness when compared with conventional antifungal treatments. Moreover, data on the safety of probiotics are still insufficient, and further research is needed.

## Background

Oral candidiasis (OC) is a fungal infection considered to be the most common oral mucosal infectious disease [1] and is mainly caused by *Candida albicans*. The detection rate of *C. albicans* in the general population is 20 to 75% [2–5]. It has also been reported that 15 to 71% of denture wearers [3, 6, 7] and 80 to 95% of HIV-infected individuals suffer from oral candidiasis [8–10]. The accepted treatment for oral candidiasis is the use of antifungal agents, such as nystatin, fluconazole, or miconazole. [11]. Because of adverse effects and side effects, such as the subsequent resistance of *candida* to antifungal agents, dysgeusia, and gastrointestinal discomfort, including nausea, vomiting and diarrhea, the clinical application of antifungal drugs can be limited [12]. Therefore, the exploration of new prophylaxis and therapeutic strategies for oral candidiasis is indicated.

Previous studies have reported that probiotics have effects on vulvovaginal candidiasis [13], dermatophytosis [14], gastrointestinal infections [15], hypertension [16] and colorectal cancer [17–19]. Known mechanisms of probiotics include regulating innate and acquired immunity and releasing antioxidants and bacteriocins to restore the balance of the microbial community and the immune system [20–22]. Meanwhile, it is also reported that probiotics are potentially promising treatment for oral diseases such as periodontal disease, dental caries, halitosis and oral candidiasis [23].

Over the last few years, probiotics have been demonstrated to enable the regulation of the oral microbiota [24]. Studies have shown that *Lactobacillus rhamnosus* [25], *Lactobacillus reuteri* [26], etc. can reduce oral *Candida* counts. However, the estimated effects of probiotics in the treatment of oral candidiasis are conflicting [25–27]. Additionally, information on the safety of probiotics is lacking. Therefore, the aim of this review is to assess the effectiveness and safety of probiotics in the prophylaxis and treatment of oral candidiasis using a meta-analysis and systematic evaluation.

## Methods

### Data sources and search strategy

This systematic review was performed according to the recommendations of the Preferred Reporting Items for Systematic Reviews and Meta-analyses (PRISMA) [28]. Two of the authors (L.J. H and M.M. Z) independently searched the following electronic databases: PubMed, EMBASE, the Cochrane Library, and Web of Science for articles published from inception to August 1, 2018. The following terms were searched in combination: (“probiotics” OR “probiotic”) AND (“oral candidiasis” OR “oral candidiases” OR “oral moniliasis” OR “oral moniliases” OR “oral candida” OR “thrush”). Manual searches of the reference and citations of the identified studies were also conducted as a supplement.

### Study selection criteria

The inclusion criteria were as follows: (I) original studies; (II) randomized controlled clinical trials or experimental mouse animal model controlled studies; (III) studies that compared probiotics (at any dosage and in any form) with control groups (placebo, blank control or other drugs); (IV) studies that reported specific outcomes of the therapeutic effect, such as the counts of *candida* and/or clinical improvement; and (V) studies published in the English language. Studies written in languages other than English, review articles, letters to the editor, meeting summaries, patented inventions, unpublished articles and articles that did not have full-text available were excluded.

### Data extraction

The two investigators (L.J. H and M.M. Z) independently identified the titles and abstracts that potentially met the inclusion criteria. Then, full-text articles were read for a complete assessment and determination of inclusion or exclusion. Each investigator independently performed the above steps. If the two review authors could not reach a consensus regarding inclusion, a third reviewer (Z.M. Y or A. Y) was invited to conduct an assessment and settle any disagreements. For each included article, data such as age, gender/sex, sample size, interventions, follow-up time and outcome indicators were extracted and summarized in a table format.

### Risk of bias of the included studies

Three investigators (L.J. H, M.M. Z, and W.W. Z) evaluated the clinical studies based on the criteria of the Cochrane handbook for systematic reviews of interventions using Review Manager 5.2 (Cochrane IMS, Oxford, UK). The considered biases were as follows: (I) random sequence generation (selection bias); (II) allocation concealment (selection bias); (III) blinding of participants and personnel (performance bias); (IV) blinding of outcome assessment (detection bias); (V) incomplete outcome data (attrition bias); and (VI) selective reporting (reporting bias).

For studies using animal models, the quality evaluation was based on the Collaborative Approach to Meta Analysis and Review of Animal Data from Experimental Studies (CAMARADES) [29]. The considered biases were as follows: (I) sample size calculation; (II) randomization of treatment or control; (III) blinded assessment of outcome; (IV) allocation concealment; (V) use of suitable animals; (VI) avoidance of anesthetics with marked intrinsic properties; (VII) statement of control of temperature; (VIII) statement of compliance with regulatory requirements; (IX) publication in a peer-reviewed journal; and (X) statement regarding possible conflicts of interest.

### Statistical analysis

Review Manager 5.2 was used to perform the meta-analyses. We assessed the therapeutic effect of probiotics on oral candidiasis by means of odds ratios (ORs) and their 95% confidence intervals (CIs) using Mantel-Haenszel statistics. Statistical heterogeneity analysis of the included studies was performed using the I^2^ metric. When I^2^ < 50%, the studies were considered to be sufficiently homogeneous, and a fixed effect model was used. In contrast, when there was heterogeneity among the studies, a random effects model was used, and sensitivity analysis was conducted to achieve homogeneity among the included studies.

For the smaller group of studies with poor homogeneity and for data provided by studies that could not be analyzed by meta-analysis, descriptive analysis and evaluation (i.e., qualitative analysis) was performed.

## Results

### Clinical research

#### Characteristics of the included studies

**Fig. 1:**
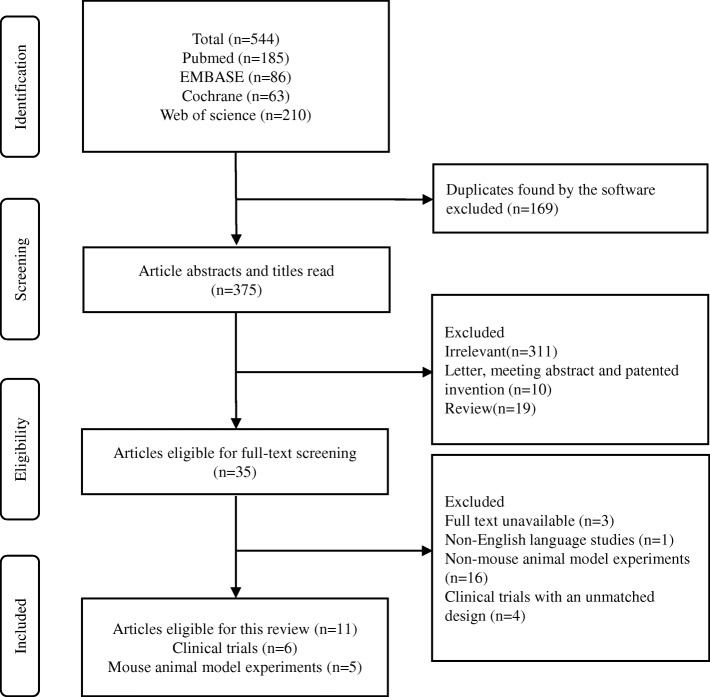
Trial flow and study selection

The number of included clinical studies was 6, which involved a total of 605 subjects (Fig. 1). The data extracted from each study are summarized in Table 1.

**Table 1:** Characteristics of the included clinical studies

Author and publication year	Probiotics group				Control group				Follow-up time (weeks)	Outcome indicators
	Age (Average)	Gender (M:F)	Sample size	Interventions	Age (Average)	Gender (M:F)	Sample size	Interventions		
Hatakka et al., 2007 [25]	58.7–95.2	36:100	92 (Baseline 136)	The combination of *L. rhamnosus* and *P. freudenreichii*	65.4–94.7	30:110	100 (Baseline 140)	Blank control	16	*Candida* counts; hyposalivation; salivary buffering capacity; dry mouth; mucosal lesions; oral pain; decayed teeth
Ishikawa et al., 2015 [27]	61.6 ± 7.8	6:24	30	The combination of *L. rhamnosus* HS111, *L. acidophilus* HS101 and *B. bifidum*	62.1 ± 9.4	8:17	25	Placebo	5	*Candida* counts
Kraft-Bodi et al., 2015 [26]	88.3 ± 5.7	35:60	84 (Baseline 95)	*L. reuteri*	87.7 ± 7.7	42:61	90 (Baseline 103)	Placebo	12	*Candida* counts; oral hygiene; gingival inflammation
Miyazima et al., 2017 [30]	66.1 ± 11.6	4:15	19	*L. acidophilus*	65.5 ± 10.5	3:17	20	Blank control	8	*Candida* counts
	61.7 ± 14.1	3:17	20	*L. rhamnosus*						
Li et al., 2014 [31]	62.72 ± 9.57	5:29	34	The combination of *B. longum*, *L. bulgaricus*, *S. thermophiles*, 2% Sodium bicarbonate solution and 2% Nysfungin cataplasm	64.84 ± 11.92	7:24	31	2% Sodium bicarbonate solution + 2% Nysfungin cataplasm	4	*Candida* counts; pain level; hyperaemia
Mishra et al., 2016 [32]	8.65	9:11	20	Probiotics mint tablet (unknown the types of probiotics)	8.90	11:9	20	0.2% Chlorhexidine digluconate	1	*Candida* counts
					9.70	12:8	20	Herbal gargle		

Four of these studies, including 480 elderly and denture wearers, compared the effects of therapy between the probiotics group and the blank or placebo group, and a meta-analysis was performed [25–27, 30]. The study by TY Miyazima et al. subdivided the probiotics group into the T1 probiotics group using *Lactobacillus acidophilus* NCFM and the T2 probiotics group using *L. rhamnosus* Lr-32 [30]. Given the existing heterogeneity of the interventions, this study combined the T1 probiotics group and T2 probiotics group as one group in the meta-analysis.

Two of these studies were excluded in the meta-analysis and were evaluated by descriptive analysis due to the comparison of probiotics with other agents. The study by Duo Li et al. evaluated the short-term effectiveness and safety of probiotics in a population aged 18–75 years by comparing a probiotics group (received topical antifungal agents—sodium bicarbonate solution and nystatin—as well as *Bifidobacterium longum*, *Lactobacillus bulgaricus* and *Streptococcus thermophilus*) with a control group (received only topical antifungal agents) [31]. The trial by Rahul Mishra et al. compared the antimicrobial effect of probiotics in children aged 6–14 years with herbal rinse and commonly used antimicrobial agents of 0.2% chlorhexidine [32]. However, the types of probiotics contained in the probiotic product were not mentioned in the article.

#### Quality of the included studies

**Fig. 2:**
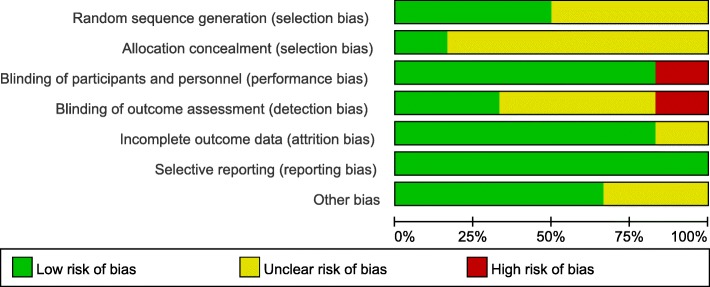
Risk of bias graph: the overall risk of each bias is presented as a percentage representing the risk in all the included studies

**Fig. 3:**
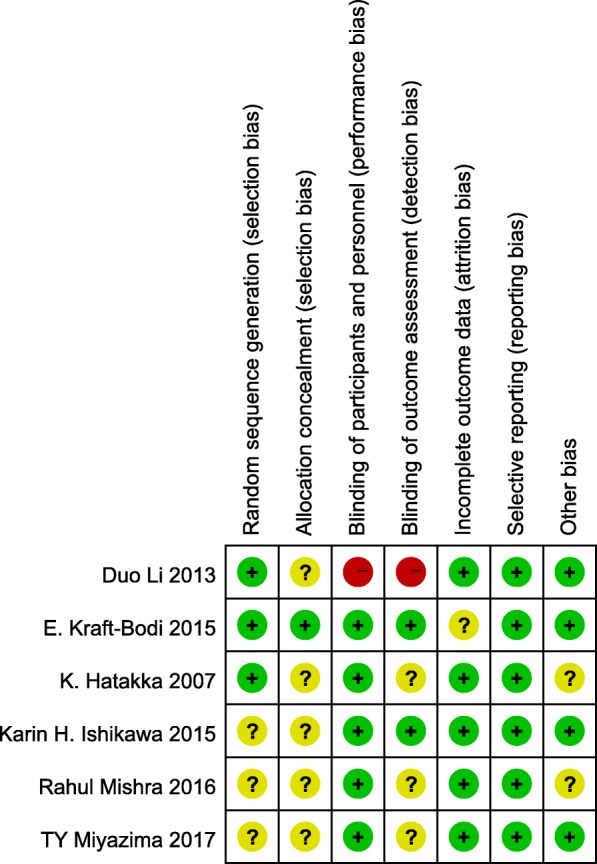
Risk of bias summary: the risk of each bias in each of the included studies is shown separately. Note: +,?, − indicate high, uncertain, and low bias, respectively

According to the Cochrane risk of bias assessment criteria, the included studies failed to achieve all seven aspects in detail. The quality evaluations of the studies are listed in Table 2. The overall risk of each type of bias is presented in Fig. 2, and the risk of each bias in each of the included studies is presented in Fig. 3. The risk of bias assessment for the included studies was conducted by 2 independent researchers (L.J. H and M.M. Z), and the consistency of the assessment results was 100%.

**Table 2:** Bias assessment of the included clinical studies

Author and publication year	Random sequence generation	Allocation concealment	Blinding of participants and personnel	Blinding of outcome assessment	Incomplete outcome data	Selective reporting	Other bias
Hatakka et al., 2007 [25]	Low risk	Uncertain	Low risk	Uncertain	Low risk	Low risk	Uncertain
Ishikawa et al., 2015 [27]	Uncertain	Uncertain	Low risk	Low risk	Low risk	Low risk	Low risk
Kraft-Bodi et al., 2015 [26]	Low risk	Low risk	Low risk	Low risk	Uncertain	Low risk	Low risk
Miyazima et al., 2017 [30]	Uncertain	Uncertain	Low risk	Uncertain	Low risk	Low risk	Low risk
Li et al., 2014 [31]	Low risk	Uncertain	High risk	High risk	Low risk	Low risk	Low risk
Mishra et al., 2016 [32]	Uncertain	Uncertain	Low risk	Uncertain	Low risk	Low risk	Uncertain

#### Effectiveness assessment

**Fig. 4:**
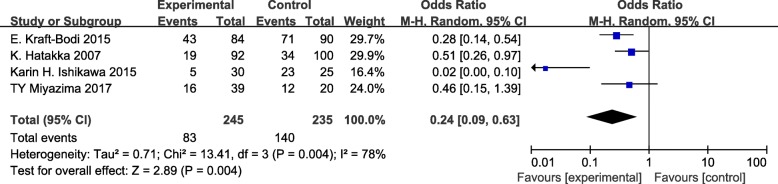
Odds ratio and 95% confidence intervals from individual studies. Forest plots evaluating the effect of probiotics (random-effect model). Note: events indicate the subjects with oral candidiasis

Meta-analysis was performed on 4 studies with a total of 480 subjects that compared a probiotics group with a placebo or blank control group [25–27, 30]. The heterogeneity analysis of these 4 studies yielded x^2^ = 13.41, *P* = 0.004, and I^2^ = 78%. The random effects model analysis showed that there was a statistically significant difference in the effect of probiotics for preventing and treating oral candidiasis in elderly and denture wearers compared with the control groups (OR = 0.24, 95% CI =0.09–0.63, *P* < 0.01; Fig. 4).

**Fig. 5:**
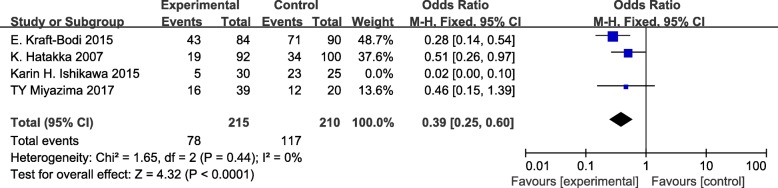
Sensitivity test: forest plot of odds ratio and 95% confidence intervals in studies at low risk of bias (fixed-effect model). Note: events indicate the subjects with oral candidiasis

Sensitivity analysis was performed in the meta-analysis. After removing 1 study with the smallest sample size, the remaining 3 studies were of good homogeneity with an I^2^ value of 0% [27]. The fixed effect model analysis showed that there was a statistically significant difference in the effect of probiotics compared with the control groups in elderly and denture wearers (OR = 0.39, 95% CI =0.25–0.60, *P* < 0.01; Fig. 5). Thus, taken together, the meta-analysis indicated that probiotics may be potentially effective for oral candidiasis in the elderly and denture wearers.

In addition, two studies compared the effect of probiotics with other drugs, including the combination of nystatin paste and a sodium bicarbonate solution, Chinese herbal rinse and 0.2% chlorhexidine digluconate rinse [31, 32]. Although hyperemia of the probiotics group using the combination of probiotics, nystatin paste and sodium bicarbonate solution improved after 4 weeks of follow-up, it did not show significant differences compared with the control group without probiotics. However, the detection rate of *Candida* spp. in the probiotics group (8.2%) was significantly lower than in the control group (34.6%) in this population aged 18–75 years (*P* = 0.038) [31]. Therefore, probiotics helped to improve the clinical symptoms of oral candidiasis and reduce the detection rate of *Candida* spp. more than using antifungal drugs alone. The 0.2% chlorhexidine digluconate rinse group had the best effects in terms of decreasing the number of *C. albicans* colony-forming units (CFUs) per milliliter in children aged 6–14 years (*P* < 0.01), followed by the probiotics group (*P* < 0.01), and the poorest outcome was from the Chinese herbal rinse (*P* > 0.01) [32]. From the above information, probiotics exhibit the potential effect of inhibiting the colonization of *Candida* on the surface of oral mucosa and improving the clinical signs and symptoms of fungal infections.

#### Safety evaluation

Only 2 of the 6 studies included information about adverse events or complaints with probiotics [26, 31]. One study reported that the probiotic had no adverse events [31], while the other reported that its most common complaints were an unpleasant taste and gastric discomfort, with incidences of 6 and 2.87%, respectively, but no severe adverse events were reported [26].

### Animal research

Through a literature search and screening, a total of 5 studies that met the criteria were included [33–37]. Table 3 lists the 5 studies and the data extracted, which included publication date, sex, age and strain of the mouse animal model, number of mice, method of *candida* infection, probiotic species, method of drug administration, intervention time, control group, and outcome indicators. The bias assessment of the included mouse animal model experiments is summarized in Table 4.

**Table 3:** Characteristics of the included animal model experiments

Author and publication year	Strain	Gender	Age (week)	Sample sizes	Method of *candida* infection	Probiotic species	Method of drug administration	Intervention time	Control group	Outcome indicators
Matsubara et al., 2012 [33]	DBA/2	Male	6–8	152	–	The combination of *L. acidophilus* and *L. rhamnosus*	0.2 ml suspension (10^9^ probiotics)	Start 2 weeks before *Candida* inoculation, qd, 27d	Blank control	*Candida* counts
									Nystatin	
Ishijima et al., 2012 [34]	ICR	Female	6	55	*Candida* cotton swab to wipe oral mucosa	*S. salivarius* K12	0.05 ml suspension	24 h, 3 h before *Candida* inoculation, 3 h, 24 h, 27 h after *Candida* inoculation	Blank control	*Candida* counts, Oral infection score, Histology
									Fluconazole	
Elahi et al., 2005 [35]	DBA/2, BALB/c (Test data not published)	Male	6–8	25	*Candida* cotton swab to wipe oral mucosa	The combination of *L. acidophilus* and *L. fermentum*	0.2 ml suspension (10^9^ probiotics)	Start 2 weeks before *Candida* inoculation, qd, 28d	Blank control	*Candida* counts, IL-4, IL-12, IFN-γ, NO
Ishijima et al., 2014 [36]	ICR	Female	6	73	*Candida* cotton swab to wipe oral mucosa	*E. faecalis*	0.05 ml suspension	24 h, 3 h before *Candida* inoculation, 3 h, 24 h, 27 h after *Candida* inoculation	Blank control	*Candida* counts, Oral infection score, Histology
									Fluconazole	
Leao et al., 2018 [37]	Wistar mice (immunosupp-ressed by intraperitoneal injection with dexamethasone (65 mg/kg))	Male	11–12	90	*Candida* cotton swab was left for 80 min under the tongue	*L. rhamnosus*	100 μL suspension (10 ^9^ cells/mL)	2 weeks before *Candida* inoculation; Probiotics intake when *Candida* inoculation	Blank control	*Candida* counts, Inflammatory infiltrate score, The cytokines levels (TNF-α, IL-1β, IL-4, IL-6, IL-10, IL-12, and INF-γ)

**Table 4:** Bias assessment of the included mouse animal model experiments

Author and publication year	Sample-size calculation	Randomization of treatment or control	Allocation concealment	Blinded assessment of outcome	Suitable animal model	Avoidance of anaesthetics with marked intrinsic properties	Statement of control of temperature	Statement of compliance with regulatory requirements	Publication in peer-reviewed journal	Statement regarding possible conflict of interest
Matsubara et al., 2012 [33]	–	✓	–	–	✓	–	–	✓	✓	–
Ishijima et al., 2012 [34]	–	✓	–	–	✓	✓	✓	✓	✓	–
Elahi et al., 2005 [35]	–	✓	–	–	✓	✓	✓	–	✓	–
Ishijima et al., 2014 [36]	–	✓	–	–	✓	✓	✓	✓	✓	✓
Leao et al., 2018 [37]	✓	–	–	–	✓	✓	✓	✓	✓	✓

Because the original data of *Candida* colony-forming units per milliliter and symptom score could not be obtained, a descriptive analysis was performed. First, compared with the blank group, *L. acidophilus*, *L. rhamnosus* and 3 × 10^9^ CFU/ml of *Streptococcus salivarius* K12 all showed the effect of reducing the *Candida* counts (*P* < 0.05) [33–35, 37]. In 2 studies conducted by Sanae A. Ishijima et al., 1.5 × 10^9^ CFU/ml and 3 × 10^9^ CFU/ml of *S. salivarius* K12 and 15 mg/ml and 30 mg/ml of *Enterococcus faecalis* all indicated an obviously significant difference from the control group (*P* < 0.01) [34, 36]. Second, *L. rhamnosus* could significantly reduce the *Candida* counts compared with nystatin (*P* < 0.05) [33]. When compared with fluconazole, a study reported that mice given 15 mg/ml of *E. faecalis* had a significant decrease in fungal burden, although this was not observed to be a complete cure [36]. These results showed that probiotics had an effect in reducing oral *Candida* counts and reducing the clinical signs and symptoms of fungal infections in animal models.

## Discussion

Attempting to treat oral candidiasis with probiotics has gradually become a topic of considerable interest in research. The clinical studies and animal model experiments included in this review reported that probiotics such as *L. rhamnosus* [25, 27, 30, 33, 37], *Propionibacterium freudenreichii* [25], *L. acidophilus* [27, 30, 33, 35], *L. reuteri* [26], *B. longum* [31], *Bifidobacterium bifidum* [27], *L. bulgaricus* [31], *S. thermophiles* [31], *L. fermentum* [35], *S. salivarius* K12 [34] and heat-killed *E. faecalis* [36] have the effect of inhibiting the excessive growth of *Candida*. Although the results of studies that met the eligibility criteria appear to demonstrate that probiotics have potential antifungal effects, the types of probiotics selected in these studies were different, and some studies focused on single probiotics [26, 30], while others focused on a combination of multiple probiotics [25, 27, 31]. For example, the study by TY Miyazima et al. reported that using *L. acidophilus* or *L. rhamnosus* alone could reduce oral *Candida* counts [30], while the study by Hatakka et al. showed that the combined use of *L. rhamnosus* and *P. freudenreichii* can reduce the risk of high yeast counts by 75% [25]. Additionally, in 2015, Ishikawa et al. indicated that the combination of probiotics containing *L. rhamnosus*, *L. acidophilus* and *B. bifidum* reduced the level of *Candida* colonization in dentures [27]. Since the types and concentrations of probiotics varied between the studies included in this review, we were unable to determine which species of probiotics and what specific doses are optimal for treating oral candidiasis. Meanwhile, research still needs to be done on which combinations of probiotics have better curative effects and how probiotics work synergistically. It is worth noting, however, that the types of probiotics in these 5 clinical studies [25–27, 30, 31] and 3 mouse-model experiments [34, 35, 37] were from the genera *Lactobacillus* and *Bifidobacterium*.

Of the included studies, there were studies comparing the effect of probiotics with conventional antifungal treatments [31, 32]. Duo Li et al. reported that oral local antifungal agents (2% sodium bicarbonate solution and nystatin) plus local probiotics helped to improve certain clinical conditions and reduce the detection rate of *Candida* spp. [31]. Moreover, a study in 2016 demonstrated that probiotic rinse was equally effective as 0.2% chlorhexidine digluconate rinse in reducing *C. albicans* counts after 1 week of intervention [32]. However, there is still insufficient evidence to prove that probiotics can completely replace antifungal agents in the treatment of oral candidiasis.

Of the 6 RCTs included, 3 evaluated the prophylaxis effect of probiotics in susceptible populations [25, 26, 32]. In total, in 366 elderly people from sheltered housing units and nursing homes, probiotics of *L. rhamnosus*, *P. freudenreichii* and *L. reuteri* were effective in reducing *Candida* counts [25, 26]. In 60 children with carious teeth (a predisposing factor of oral *Candida* carriage [38]), the prophylactic effect of probiotics on oral candidiasis was revealed [32]. The possible prophylactic mechanisms included competition with pathogenic microorganisms for nutrients and receptors [39, 40] and releasing external metabolites and producing hydrogen peroxide (H_2_O_2_), antagonizing the excessive growth of *Candida* [41, 42]. Based on current clinical studies, it is indicated that the prophylactic use of probiotics might potentially reduce the mobility of oral candidiasis and thus decrease the economic burden of the disease.

In addition to commonly used probiotic species, an animal experiment included in this review reported that heat-killed *E. faecalis* had an immunostimulatory effect in a murine model of oral candidiasis, which is beneficial for the treatment of oral candidiasis [36]. Heat-treated *E. faecalis* has been reported to have immunoenhancing effects that include increasing cell-mediated immunity, humoral immunity, monocyte/macrophage function, and natural killer cell activity in nonsensitized mice [43]. In 2012, an animal study aimed at the lysed *E. faecalis* in the murine model of allergic rhinitis suggested that *E. faecalis* has an immunoregulatory activity [44]. In a pilot study, living nonpathogenic *E. faecalis* was shown to be beneficial for the treatment of asthma [45]. However, no study has investigated the clinical effect of *E. faecalis* on oral candidiasis. This might open up a new research direction for the study of probiotics.

With respect to safety, the adverse events of probiotics reported in the in vivo studies included in this systematic review were gastrointestinal discomfort and unpleasant taste [26]. No severe adverse events were reported from either the clinical trials or the animal studies. However, a report issued by the Agency for Healthcare Research and Quality (AHRQ) in 2011 concluded that although the existing clinical trials do not indicate an increased risk, this does not necessarily confirm the safety of probiotics in intervention studies with confidence [46]. The theoretically possible side effects of probiotics were systemic infections, deleterious metabolic activities, excessive immune stimulation in susceptible individuals, and gene transfer [47]. For instance, a study reported that a newborn with an umbilical bulge developed sepsis after a 10-day administration of *Bifidobacterium breve* BBG01 [48]. In contrast, a study published in 2015 indicated that probiotics are generally safe for most populations based on the preponderance of the data from clinical trials, animal studies, and in vitro studies [49]. In another study, 80 children aged 3 months to 3 years old with rotavirus diarrhea were divided into placebo and treatment groups. The treatment group given commercial sachets of *Bifidobacterium* did not report adverse events during or after treatment [50]. Moreover, *Lactobacillus*, *Bifidobacterium*, and *Enterococcus* have been used as food additives for a long period of time [51]. It has been demonstrated that the widespread use of beverages containing probiotics such as *Lactobacillus* and *Bifidobacterium* can reduce the prevalence of oral candidiasis in healthy individuals [52]. Thus, the reports are contradictory. Therefore, further research is still needed to confirm the safety and to evaluate adverse events related to probiotics in healthy people or patients.

Although aimed at collecting the best evidence to date, this review still had limitations. First, the inconsistency of the evaluation criteria of the clinical effects among studies might be a source of heterogeneity. The limited number of trials and subjects was also a restriction. More trials are needed to verify the results above. Second, the standard for the prophylactic and therapeutic use of probiotics has not been established, and the combination of probiotics, dosages, dosing regimens, vehicles, adverse reactions, biodynamics and cost-effectiveness of the probiotics also need to be determined. Furthermore, although probiotics are beneficial to humans, as a living biological agent, it is still necessary to consider biological tolerance and whether probiotics are suitable for various types of people, such as immunosuppressed patients, infants and pregnant women. Much still needs to be learned about this new treatment for oral candidiasis.

## Conclusions

It is concluded in this systematic review that probiotics were significantly superior to the placebo and blank control in preventing and treating oral candidiasis both in clinical trials of elderly and denture wearers and in animal experiments, including inhibiting the colonization of *Candida* on the surface of oral mucosa and reducing the clinical signs and symptoms of fungal infections. However, although probiotics showed a favorable effect in treating oral candidiasis, more evidence is required to confirm their effectiveness when compared with conventional antifungal treatments. Moreover, although the commonly reported adverse events of probiotics were relatively mild, the evidence for safety is still insufficient, and further research is needed.

## Data Availability

All data generated or analyzed during this study are included in this published article.

## Abbreviations

CI: Confidence interval
EMBASE: Excerpta medica database
F: Female
h: Hours
M: Male
OR: Odds ratio
